# Identification of the functional domain of the dense core vesicle biogenesis factor HID-1

**DOI:** 10.1371/journal.pone.0291977

**Published:** 2023-09-26

**Authors:** Blake H. Hummer, Theodore Carter, Breanna L. Sellers, Jenna D. Triplett, Cedric S. Asensio

**Affiliations:** Department of Biological Sciences, University of Denver, Denver, CO, United States of America; Normandie Universite, UNITED STATES

## Abstract

Large dense core vesicles (LDCVs) mediate the regulated release of neuropeptides and peptide hormones. HID-1 is a trans-Golgi network (TGN) localized peripheral membrane protein contributing to LDCV formation. There is no information about HID-1 structure or domain architecture, and thus it remains unknown how HID-1 binds to the TGN and performs its function. We report that the N-terminus of HID-1 mediates membrane binding through a myristoyl group with a polybasic amino acid patch but lacks specificity for the TGN. In addition, we show that the C-terminus serves as the functional domain. Indeed, this isolated domain, when tethered to the TGN, can rescue the neuroendocrine secretion and sorting defects observed in HID-1 KO cells. Finally, we report that a point mutation within that domain, identified in patients with endocrine and neurological deficits, leads to loss of function.

## Introduction

The regulated release of polypeptides is essential for many biological processes, such as feeding, sleep, and physiological homeostasis [1–3]. Secretion of peptide hormones and neuropeptides depends on their efficient sorting and concentration into large dense core vesicles (LDCVs) or secretory granules capable of regulated exocytosis. LDCVs bud from the trans-Golgi network (TGN), where their soluble cargoes, such as the members of the granin family, aggregate under the mild acidic pH and redox conditions to begin to form a dense core. Budded vesicles then undergo a series of maturation steps including homotypic fusion, acidification, propeptide processing, and removal of missorted soluble and transmembrane proteins [4–10]. Despite the importance of LDCVs, the exact mechanisms controlling their formation remain poorly understood.

Recently, several studies have shown that the formation of LDCVs relies on a variety of cytosolic factors. Rab2 and its interactors orchestrate cargo sorting to LDCVs [11–17]. The BAR (bin/amphiphysin/Rvs) domain containing proteins ICA69 and PICK1 heterodimerize to regulate formation and/or maturation of LDCVs [18,19] and Arfaptin-1 regulates insulin LDCV fission [20]. AP-3 and VPS41 contribute to transmembrane protein sorting to the regulated secretory pathway [21–23] and BAIAP3 influences LDCV maturation by controlling endosomal recycling of LDCV transmembrane proteins [24]. HID-1 was identified by a forward genetic screen looking for defects in neuropeptide sorting in *C*. *elegans*. Worms lacking HID-1 display reduced levels of LDCV soluble cargo and impaired neurosecretion [25,26]. Interestingly, HID-1 localizes to the TGN and its expression seems restricted to professional secretory cells, such as endocrine or neuroendocrine cells [27], suggesting that it might contribute to LDCV biogenesis. Consistent with this, mice lacking HID-1 specifically in pancreatic β-cells are glucose intolerant and display impaired insulin secretion [28]. In neuroendocrine PC12 cells, HID-1 modulates LDCV cargo sorting, presumably through regulation of TGN pH [29].

Initial bioinformatic analysis of HID-1 amino acid sequence predicted the presence of several transmembrane spanning domains [30]. However, subsequent work demonstrated that HID-1 behaves as a peripheral membrane protein that associates with the TGN from the cytosol [27]. Besides the presence of an N-myristoyl, HID-1 has no predicted quintessential lipid binding domains, protein-protein interacting domains, or any other known domains, and thus the mechanism by which it associates with the TGN and performs its function remains completely unknown.

Here we show that an N-myristoyl and a polybasic patch within the first 17aa of the N-terminus of HID-1 are required to increase its affinity for membranes but fail to provide TGN specificity. We further demonstrate that the highly conserved C-terminus serves as the functional domain of HID-1. We show that artificial tethering of this domain to the TGN is sufficient to rescue the phenotype observed in HID-1 KO PC12 cells. Finally, we report that a point mutation within that domain, identified in patients with endocrine and neurological deficits [31], leads to loss of function.

## Materials and methods

### Molecular biology

All constructs (**S1 Table**) were verified by Sanger sequencing (Quintarabio). Primers were obtained from IDT and restriction enzymes from NEB. HID-1 lentiviral plasmid was generated by amplifying HID-1 from rat PC12 cell cDNA using the following primers: P1_F and P1_R. The main isoform amplified from these cells corresponds to isoform #2, which has been reported for mouse (Uniprot: Q8R1F6-2) and for human (Uniprot: Q8IV36-2). The PCR products were then subcloned into pLenti-puro using the restriction enzymes XbaI and BstEII. The G2A and Δ22 HID-1 mutant plasmids were constructed by amplifying from the WT HID-1 pLenti-puro plasmid using the following primers: G2A Forward: P2_F and P2_R; P3_F and P3_R. The PCR products were subcloned into pLenti-puro using the restriction enzymes XbaI and BstEII. HID-1 C-terminus human mutation was amplified from WT HID-1 and cloned in pLenti-puro by Gibson using the following primers: P4_F and P4_R. The GFP-TGN38-HID-1 C-terminus fusion was made by amplifying GFP-TGN38 from a premade plasmid using the primers: p5_F and P5_R. The HID-1 C-terminus was amplified from WT HID-1 using the primers: P6_F and P6_R. These PCRs were cloned into the pCAGG vector by Gibson. HID-1-GFP tail fusions were amplified with the following primers: pEGFP-N1-HID-1-N22: P7_F and P7_R; pEGFP-N1-HID-1-N22(G2A): P8_F and P8_R; pEGFP-N1-HID-1-N17: P9_F and P9_R; pEGFP-N1-HID-1-N17(FVL): P10_F and P10_R; pEGFP-N1-HID-1-N17(KRK): P11_F and P11_R. PCRs were subcloned into pEGFP-N1 using BamHI and AgeI. The HID-1 truncation constructs were amplified using the following primers: FUGW-HID-1(1–583)-HA: P14_F and P14_R; FUGW-HID-1(1–686)-HA: P15_F and P15_R. PCRs were subcloned into FUGW or pLenti-puro using Gibson reaction. pEGFP-C3 plasmid was digested with XhoI and BamHI and then Gibson assembled using TGN38-HID-1 C-terminus (HID-1^687−788^) gene block (Twist Bioscience).

### Cell culture and lentivirus production

Rat INS-1 cells, originally obtained from the laboratory of Dr. Peter Arvan (University of Michigan), were maintained in RPMI supplemented with 1mM sodium pyruvate, 10% fetal bovine serum and 50μM β-mercaptoethanol under 5% CO_2_ at 37°C. Rat PC12 cells, originally obtained from the laboratory of Dr. Regis Kelly (UCSF), were maintained in DMEM supplemented with 10% horse serum and 5% calf serum under 5% CO_2_ at 37°C. Transfection of PC12 and INS-1 cells was performed using Fugene HD (Promega) or Lipofectamine 2000 (Invitrogen) according to the manufacturer’s instructions. HEK293T cells were maintained in DMEM with 10% fetal bovine serum under 5% CO_2_ at 37°C. Lentivirus was produced by transfecting HEK293T cells with FUGW or pLenti-puro, psPAX2, and pVSVG using PEI at 1μg/μL in final reaction volume or Fugene HD according to the manufacturer’s instructions. 24hr after virus application, stably transduced cells were then selected for ~24hr using 3ug/mL puromycin.

### CRISPR/Cas9

The human codon-optimized Cas9 and chimeric guide RNA expression plasmid (pX330) developed by the Zhang lab were obtained from Addgene [32]. To generate gRNA plasmids, a pair of annealed oligos (20 base pairs) was ligated into the single guide RNA scaffold of pX330. The following gRNAs sequences were used: Forward2: 5′-CACCGAGCCACCGACAATGCTTTCT-3′; Reverse2: 5′-AAACAGAAAGCA-TTGTCGGTGGCTC-3′ to generate HID-1 KO. The following primers were used to genotype HID-1 KO cells: P1F: 5′-GCACTAAACGGGGAGTCC-3′; P1R 5′-GCATAATGGACACTGAAGGTAGGAGG-3′; P2F: 5′-GGCATGGACGAGCTGTACAAG-3′; P2R: 5′- CCAGTCCACTGGGATGGC-3′; P3F: 5′-GTGTAACTCTGGCTACTCGATTCC-3′; P3R: 5′-CTTGTAGCACAGGGTGGCC-3′. To test for the presence of indels, primers P3F/P3R were used. The resulting PCR products were ligated into pBluesecript II KS. Isolated plasmids from 24 random colonies were then analyzed for the presence of indels by sequencing.

### Flow cytometry

INS-1 HID-1 KO cells were transfected with either GFP, N22-GFP, G2A-GFP, N17-GFP, N17FVL-GFP, or N17KRK-GFP. Transfected cells were either untreated or treated for 50 seconds in 0.1% saponin in PBS and then immediately fixed with 4% paraformaldehyde. Quantitative analysis of fluorescence was performed using flow cytometry to determine the percent decrease in cells with a positive GFP signal in saponin-treated cells compared to untreated cells.

### Antibodies

HA (3F10) rat mAb was obtained from Roche, SgII rabbit antibody from Meridian Life Science (USA), TGN38 mouse mAb from BD Biosciences (USA), tubulin and actin mouse mAb from Developmental Hybridoma Bank Studies (USA), HID-1 mouse mAb from Novus Biologicals (USA), GFP mouse mAb from Proteintech (USA), and goat anti-rabbit Alexa Fluor 647, anti-mouse Alexa Fluor 488, anti-mouse Alexa Fluor 647, and anti-rat Alexa Fluor 488 secondary antibodies from ThermoFisher (USA).

### Secretion assays

PC12 cells were plated on poly-l-lysine, washed, and incubated in Tyrode’s buffer containing 2.5 mM K^+^ (basal) or 90 mM K^+^ (stimulated) for 30 min at 37°C. The supernatant was then collected, cell lysates prepared as previously described [33], and the samples analyzed by quantitative fluorescence immunoblotting. For transient transfection experiments, HID-1 KO PC12 cells were plated on poly-l-lysine, transfected with 200ng NPY-sfCherry3 in addition to 500ng HID-1-GFP, GFP-TGN38, or GFP-TGN38-HID-1 C-terminus. 48 hours after transfection, cells were washed and incubated in Tyrode’s buffer containing 2.5 mM K^+^ (basal) or 90 mM K^+^ (stimulated) for 30 min at 37°C. The supernatant was collected and cell lysates prepared as described above. The amount of cellular and secreted NPY-sfCherry3 was determined using a plate reader (Tecan).

### Immunofluorescence and confocal microscopy

PC12 and INS-1 cells were rinsed with PBS and fixed in 4% paraformaldehyde in PBS and incubated for 20 min at room temperature followed by a 20min quenching step with 100mM NH_4_Cl in PBS. For saponin extraction experiments, prior to fixation, cells were treated with 0.1% saponin in PBS for 50s and washed with PBS. Cells were blocked in PBS containing 2% BSA, 1% fish skin gelatin, and 0.02% saponin. Primary antibodies were diluted in blocking solution at 1:1000 (TGN38), 1:1000 (SgII) and 1:500 (HA.11). The secondary goat anti-rat and donkey anti-mouse antibodies were diluted in blocking solution at 1:1000. Images were collected with a 63× objective (Oil Plan Apo NA 1.49) and an ImageEM X2 EM-CCD camera (Hamamatsu, Japan). For quantification of SgII, Z-stack were converted into a max intensity projection. ROIs were drawn around cells in the GFP channel which were then used to determine the background subtracted mean fluorescence of SgII in these GFP positive cells.

### Fluorescence loss in photobleaching

INS-1 HID-1 KO cells stably expressing HID-1-GFP or G2A-GFP were transfected with sialyltransferase-pTAG-RFP657. The following day, transfected cells were plated on poly-L-lysine coated coverslips. Two days after transfection, coverslips were placed in an open imaging chamber (Thermofisher) and imaged using an Olympus Fluoview scanning confocal microscope and a 63× oil objective (NA 1.42) at a resolution of 512 × 512 pixels with a sampling speed of 8.0 μs/pixel with Kalman filter (integration count 5). The region of interest for photobleaching was selected based on proximity to the TGN and was positioned at the periphery of the cell. This was to ensure our primary objective of specifically photobleaching the cytosolic pool was met, while minimizing the risk of inadvertently affecting the Golgi-bound pool during the experiment. Cells were bleached for ~1sec at 100% laser output and imaged followed by a 15sec recovery period. Movies were taken for a total of 5min. Analysis of fluorescence intensity was restricted to the Golgi area by using the sialyltransferase-pTAG-RFP657 to generate an ROI within ImageJ.

### Statistics

Unless indicated otherwise, all statistical analysis was performed using the two-tailed Student’s *t* test. Statistical analyses were conducted using Excel or Prism.

### Figure preparation

Images were processed using ImageJ; any changes in brightness and contrast were identical between samples meant for comparison.

## Results and discussion

### HID-1 N-terminus mediates membrane binding but is not strictly required for TGN localization

**Fig 1:**
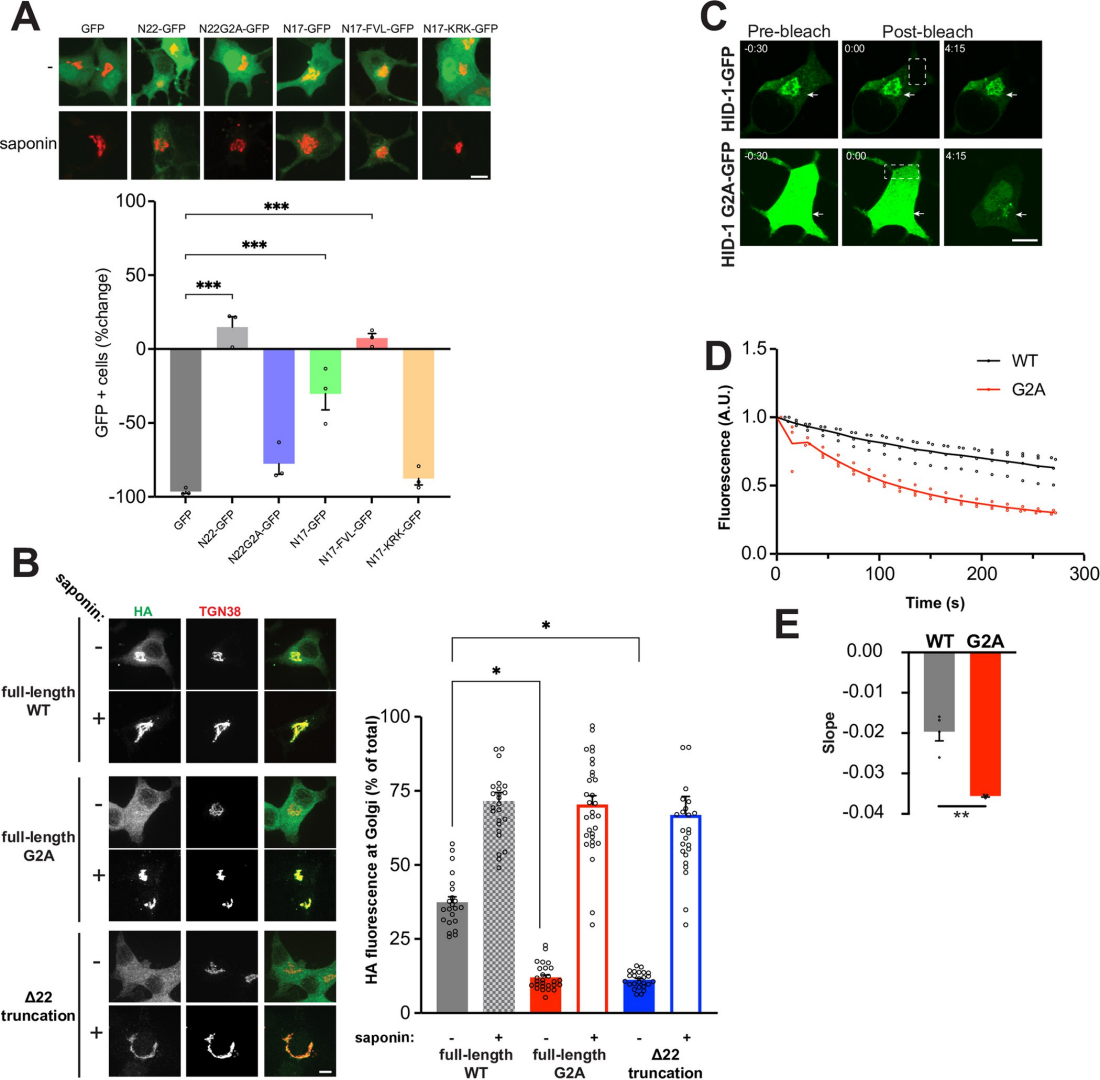
HID-1 N-terminus mediates membrane binding but is not strictly required for TGN localization. (**A**) INS-1 cells were co-transfected with sialyltransferase-RFP657 and indicated GFP constructs. Cells treated or not with saponin were fixed and imaged. Quantification of the number of GFP positive cells was performed using flow cytometry and data represent the percentage changes in the number of GFP positive cells after saponin treatment compared to untreated cells (>3000 cells per experiment). ***, p<0.001 relative to GFP by one-way ANOVA (n = 3 independent transfections). The bar graphs indicate mean ± standard error (s.e.) (**B**) INS-1 cells were transfected with WT, G2A or Δ22 HID-1-HA and treated or not with saponin before fixation and immunostained for HA and TGN38. TGN-localized and total HA immunoreactivity was quantified and expressed as a ratio. *, p < 0.05 relative to WT by one-way ANOVA (n = 22 cells for WT and n = 25 cells for WT + saponin; 27 cells for G2A and n = 31 cells for G2A + saponin; n = 25 cells for Δ22 and n = 25 cells for Δ22 + saponin). (**C-E**) INS-1 HID-1 KO cells stably expressing WT or G2A HID-1-GFP were transiently transfected with sialyltransferase-RFP657 and analyzed by fluorescence loss in photobleaching. The white boxes represent the defined photobleaching region of interest (ROI). Arrows designate the TGN (determined by co-transfected sialyltransferase-RFP657—not shown). Time (0:00) indicates the timeframe immediately after the first photobleaching pulse. Quantification of the change in fluorescence at the TGN is shown in (**D**) and of the initial decay slope in (**E**). **, p<0.01; WT n = 4, G2A n = 3 independent sialyltransferase-RFP657 transfections. Scale bar indicates 10μm. The bar graphs indicate mean ± s.e.

We have previously generated HID-1 KO PC12 cells using a genome-editing strategy that led to the fusion of tdTomato in frame directly after the first 22 amino acids (N22) of HID-1 followed by an early stop codon [29]. Interestingly, we noticed that the signal of our fluorescent reporter was not purely cytosolic but rather perinuclear, suggesting that the HID-1 N-terminus might be sufficient for membrane binding. As HID-1 is N-myristoylated [34], we focused on this part of the protein first. INS-1 cells are bigger, flatter, and more amenable to imaging than PC12 cells, thus we have relied on INS-1 cells for imaging experiments and PC12 cells for functional experiments for the remainder of this study. We transfected INS-1 cells with N22 fused to GFP (N22-GFP) and compared its behavior to cells transfected with GFP alone as a control. Whereas GFP alone washed away completely during saponin treatment pre-fixation as expected for a cytosolic protein, we found that a pool of N22-GFP was resistant to cytosolic extraction, suggesting membrane binding (**Fig 1A**). We next tested the role of N-myristoylation by generating a G2A N22 mutant fused to GFP (N22G2A-GFP) and found that it behaved similarly to cytosolic GFP, suggesting decreased membrane affinity (**Fig 1A**).

Typically, N-myristoylation per se is not sufficient for membrane targeting and only promotes weak interaction with the membrane. This type of lipidation is thus often found in combination with other motifs or modifications [35]. In some cases, the presence of additional lipid modifications, such as palmitoylation, increases membrane binding affinity and specificity, but analysis of the HID-1 N22 sequence failed to identify additional predicted lipidation sites. Alternatively, the myristoyl group can be found in association with polybasic amino acid patches [35], and HID-1 has three conserved basic residues in its first 22 amino acids (**S1A Fig**). In other cases, the myristate moiety increases membrane affinity by rendering an N-terminal alpha-helix more amphipathic, as it has been shown for both Arf and Arl proteins [35,36]. Of note, the first 17 amino acids of HID-1 are predicted to fold as an alpha-helix, and this fragment (N17) behaved similarly to N22-GFP (**Fig 1A**). To distinguish between the two possibilities, we reasoned that replacement of positively charged amino acids with alanine (K7A, R11A, K12A; N17KRK) should affect both motifs, whereas substitution of hydrophobic residues for polar residues (F10Q, V14Q, L17Q; N17FVL) should only affect a putative amphipathic helix. Strikingly, the N17KRK mutant washed away completely during cytosolic extraction, whereas the N17FVL construct was resistant to saponin extraction (**Fig 1A**). To quantify these observations, we repeated the experiment to determine the percent change in the number of GFP positive cells in presence or absence of saponin by flow cytometry. Consistent with our qualitative assessment, we observed little to no change in the percent of GFP positive cells transfected with N22-GFP, N17-GFP, or N17FVL-GFP. Conversely, the large majority of cells transfected with GFP alone, N22G2A-GFP, or N17KRK-GFP lost their fluorescence signal with saponin treatment (**Fig 1A**). We obtained similar results in PC12 cells (**S1B Fig**). Altogether, these results suggest that a myristoyl group and a polybasic patch within the first 17 amino acids of the N-terminus of HID-1 contribute to membrane binding.

Although the N-terminus of HID-1 is capable of membrane binding, our reporter did not show enrichment at the TGN as evidenced by the lack of co-localization with a co-transfected TGN marker (sialyltransferase-TagRFP657) (**Fig 1A**). To directly test whether the N-terminus is required for HID-1 localization, we generated HID-1 KO INS-1 cells using CRISPR/Cas9. As there are currently no commercially available antibodies for HID-1 western blotting, we validated our HID-1 KO by indel sequencing and immunofluorescence (**S2 Fig**). We then used lentivirus to stably reintroduce HA tagged wildtype (WT), myristoylation deficient (G2A), or N-terminal deletion (Δ22) versions of HID-1. Although the G2A and Δ22 mutants displayed a greater amount of cytosolic signal at steady-state when compared to WT HID-1, pre-fixation saponin extraction revealed the presence of a pool co-localizing with a TGN marker (**Fig 1B**). These observations suggest that the N-terminus of HID-1 increases its affinity for membrane but is not strictly required for TGN localization.

To further investigate these findings, we conducted a series of experiments relying on fluorescence loss in photobleaching (FLIP). FLIP provides information about dynamics of fluorescently labeled molecules in various cellular structures. In our case, we reasoned that photobleaching the cytosolic pool of HID-1 while measuring the change in HID-1 signal at the TGN should enable us to quantify the difference in binding behavior of WT vs G2A HID-1. Using INS-1 cells expressing WT or G2A HID-1-GFP, we selected defined cytosolic regions at the periphery of cells and continuously (every 15s) photobleached the HID-1-GFP molecules undergoing passive diffusion within said area. We concomitantly determined HID-1-GFP fluorescence over time at the TGN determined by co-transfected sialyltransferase-RFP657. Comparison of WT and G2A HID-1-GFP confirmed that the absence of the myristoyl moiety only partially decreased HID-1 TGN immobile pool from 66.3±4.6% for WT to 30.3±3.1% for G2A HID-1-GFP and led to a faster cycling off the TGN as evidenced by analysis of the initial decay slope between WT and G2A (**Fig 1C–1E**).

### HID-1 myristoylation is not required for function

**Fig 2:**
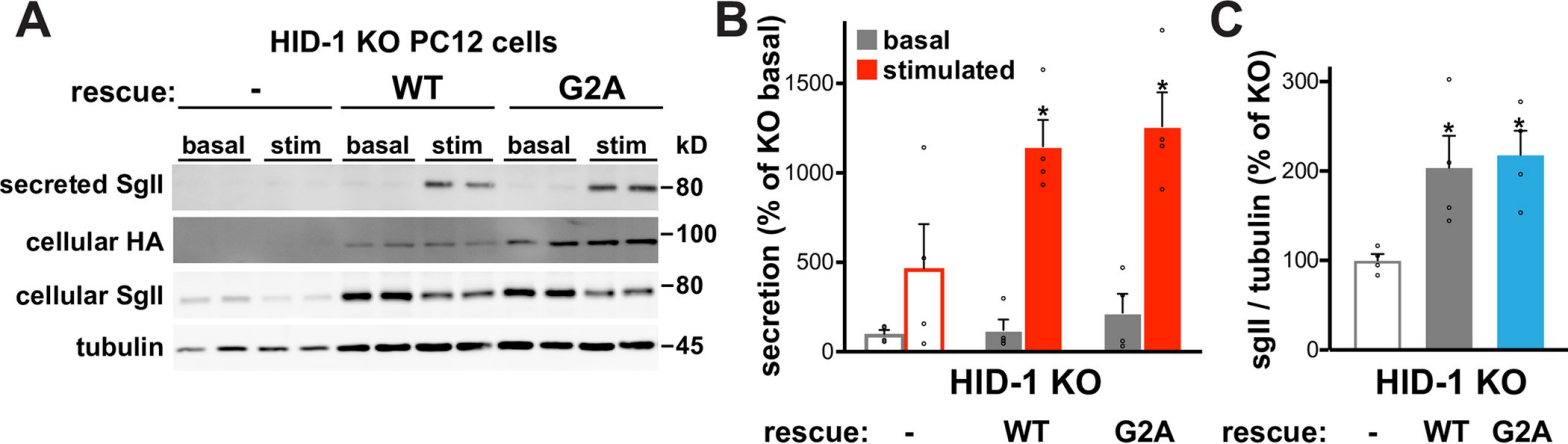
HID-1 myristoylation is not required for function. (**A-C**) HID-1 KO PC12 cells transduced with indicated HID-1-HA lentivirus were washed and incubated for 30 minutes in Tyrode’s solution containing 2.5 mM K^+^ (basal) or 90 mM K^+^ (stimulated). Cellular and secreted SgII were measured by quantitative fluorescent immunoblotting (**A**), with the secreted SgII normalized to tubulin and expressed as percent of basal secretion in the KO (**B**), and the cellular SgII normalized to tubulin (**C**). *, p < 0.05 relative to HID-1 KO by one-way ANOVA (n = 4). The bar graphs indicate mean ± s.e.

We have previously observed that loss of HID-1 in PC12 cells significantly reduced basal cellular levels of the LDCV soluble neuropeptide secretogranin II (SgII) and impaired the stimulation of SgII release by depolarization [29]. To determine whether the residual TGN pool observed for G2A HID-1 is functional, we tested its ability to rescue SgII levels and secretion caused by the loss of HID-1. For this, we first generated PC12 HID-1 KO using CRISPR/Cas9 (**S2 Fig**) and overexpressed WT or G2A HID-1-HA using lentivirus in HID-1 KO cells. We found that under conditions of overexpression G2A was indeed sufficient to fully rescue the defect in SgII storage and secretion (**Fig 2A–2C**). While we have not tested the rescue ability of G2A at endogenous levels, these data suggest that myristoylation increases membrane affinity, but it is not an absolute requirement for HID-1 function. It further indicates that an additional domain within HID-1 is responsible for its function.

### The C-terminus of HID-1 serves as the functional domain

**Fig 3:**
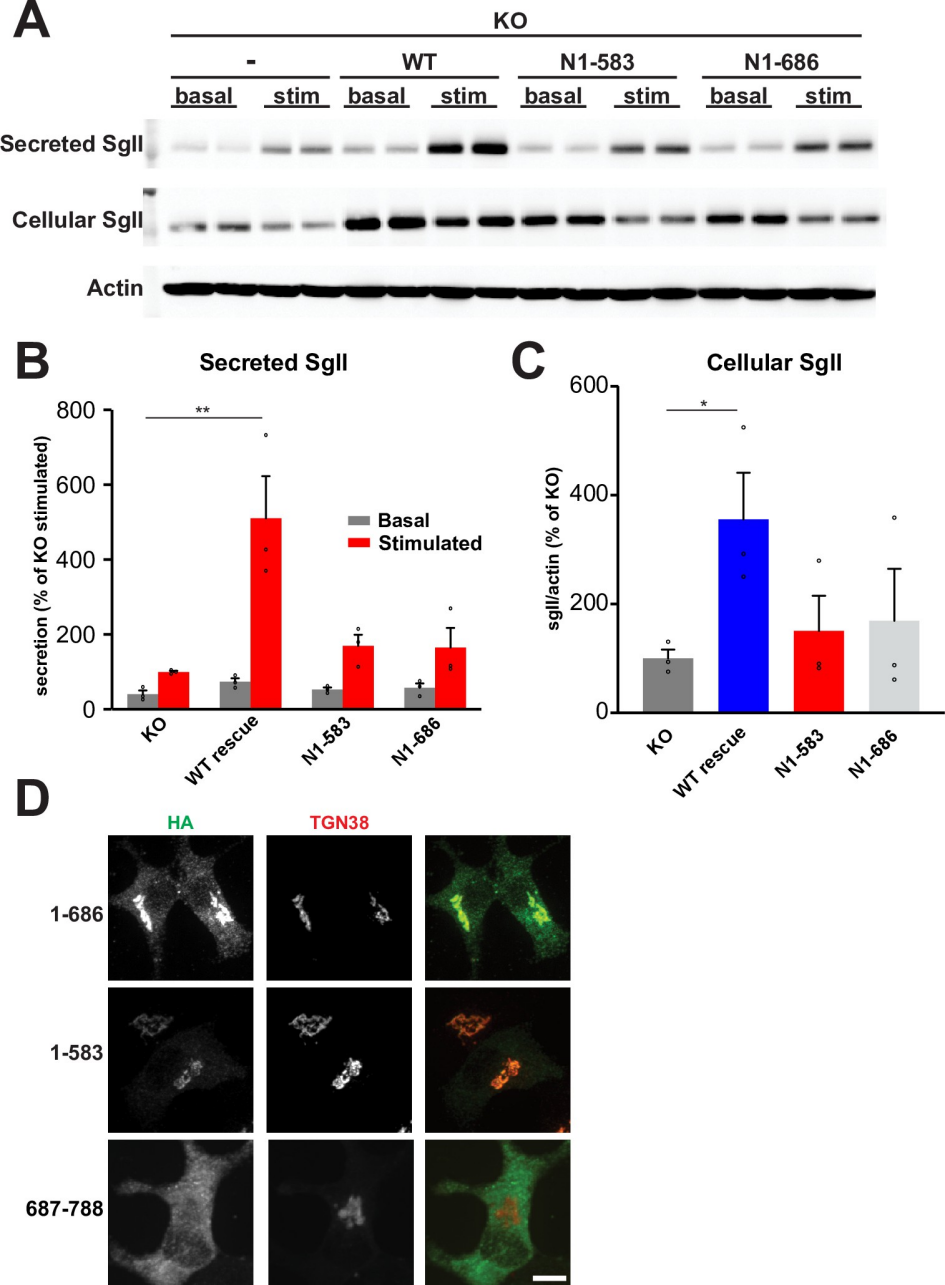
The highly conserved C-terminus of HID-1 serves as the functional domain. (**A**) Secretion assays were performed using HID-1 KO PC12 cells stably expressing WT HID-1 or indicated HID-1 truncations. Cellular and secreted SgII were measured by quantitative fluorescent immunoblotting as described in **Fig 2**, with the secreted SgII normalized to actin and expressed as percent of basal secretion in the KO (**B**), and the cellular SgII normalized to actin (**C**). *, p < 0.05; **, p < 0.01 relative to KO (n = 3) by one-way ANOVA. The bar graphs indicate mean ± s.e. (**D**) HID-1 KO INS-1 cells were stably transfected with indicated constructs. Cells were fixed, immunostained for HA and TGN38, and imaged using a spinning disk confocal. Scale bars indicate 10μm.

The C-terminus of HID-1 (amino acids 686–788) displays a particularly high degree of conservation across species (**S3 Fig**) and is preceded by a predicted disordered region (amino acids 584–788). We hypothesized that the C-terminus with or without this disordered region might act as the functional domain. To test this directly, we stably expressed HA-tagged C-terminal truncations (1–686 and 1–583) in HID-1 KO PC12 cells for functional assays and found that they both failed to rescue the defect in SgII regulated secretion (**Figs 3A–3C and S4**). In the case of proteins that are not purely cytosolic, such as peripheral membrane proteins, a lack of functional rescue by mutants could be an indirect consequence of their inability to localize to the proper compartment (TGN in the case of HID-1). However, we observed that, when these same C-terminal truncations were stably expressed in HID-1 KO INS-1 cells and immunostained for HA, they still localized to the TGN (**Fig 3D)**. These data indicate that HID-1 C-terminus is required for function.

**Fig 4:**
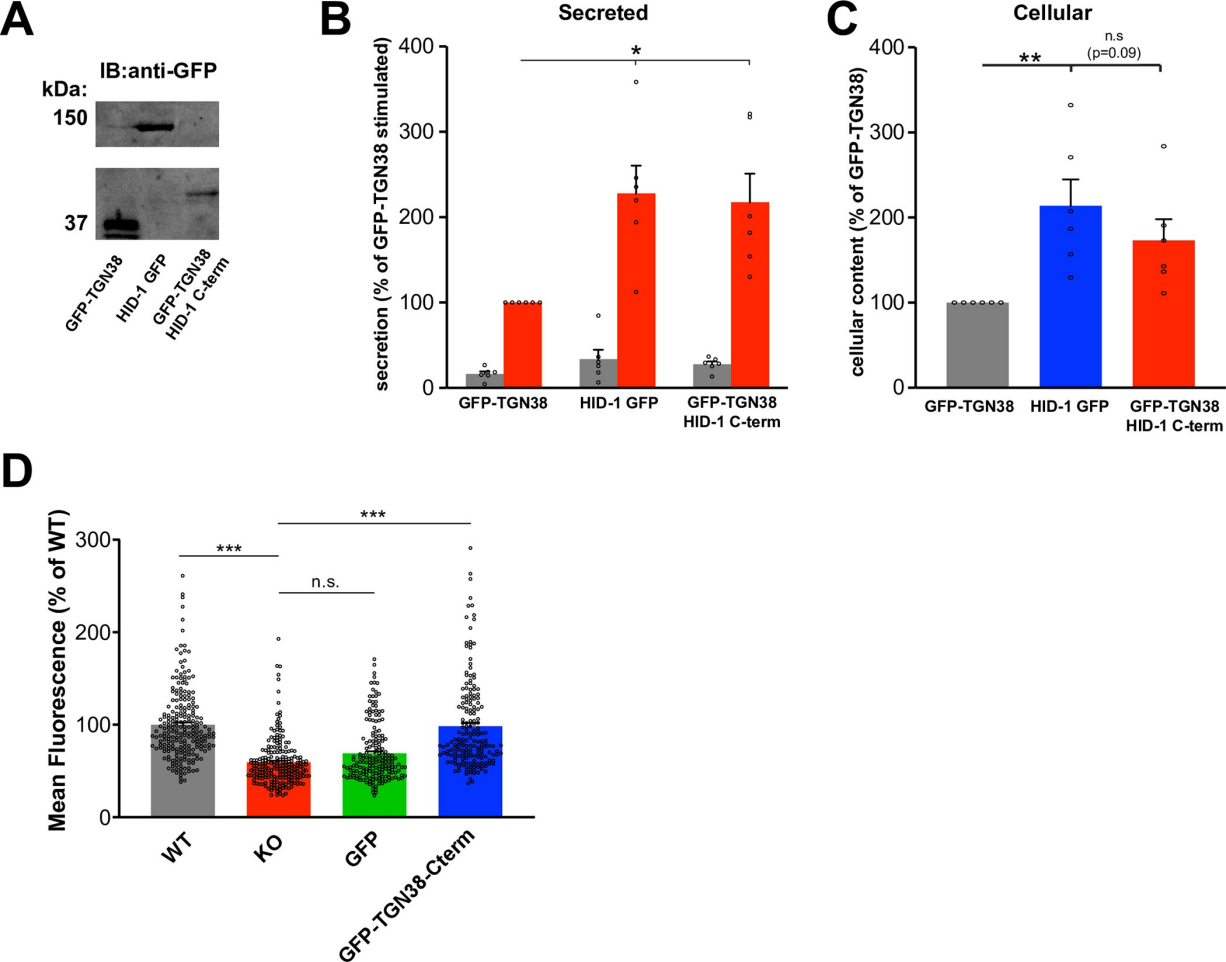
Artificial tethering of the HID-1 C-terminus domain to the TGN is sufficient to rescue the HID-1 KO phenotype. (**A-C**) HID-1 KO PC12 cells were transiently co-transfected with GFP-TGN38, GFP-TGN38-HID-1-C-terminus or HID-1-GFP and NPY-sfCherry3. **(A)** Anti-GFP immunoblot showing expression level of the fusion proteins. (**B-C)** Cells were washed and incubated in Tyrode’s buffer containing 2.5 mM K^+^ (basal) or 90 mM K^+^ (stimulated) for 30 min at 37°C. The amount of cellular and secreted NPY-sfCherry3 was determined using a plate reader (Tecan). *, p < 0.05, **, p < 0.01 relative to GFP-TGN38 by one-way ANOVA (n = 6). The bar graphs indicate mean ± s.e. (**D**) HID-1 KO PC12 cells were transiently co-transfected with GFP, GFP-TGN38-HID-1-C-terminus, immunostained for SgII and the amount of fluorescence in GFP positive cells was quantified and normalized to WT PC12 cells. ***, p<0.001 relative to HID-1-KO by one-way ANOVA (n = 226 cells for WT, n = 217 cells for KO, n = 186 cells for GFP, n = 185 cells for GFP-TGN38-Cterm from three independent experiments). The bar graphs indicate mean ± s.e.

We next tested if the C-terminal domain (amino acids 687–788) can be sufficient for function by itself. As stable expression of this HA-tagged domain in HID-1 KO INS-1 cells revealed a diffuse distribution across the cells without obvious enrichment at the TGN (**Fig 3D**), we decided to artificially tether this domain to the TGN. For this, we fused HID-1^687−788^ (HID-1 C-terminus) to the C-terminus of GFP-TGN38 to constitutively localize it to the TGN. Transient expression of this fusion protein in HID-1 KO PC12 cells increased regulated secretion of a co-transfected regulated secretory cargo (NPY-sfCherry) compared to GFP-TGN38. The extent of the rescue observed for the C-terminus construct was similar to that of full-length HID-1-GFP (**Fig 4A–4C**). Although the cellular content of NPY-sfCherry tended to be higher in cells expressing the C-terminal domain, it did not reach significance (p = 0.09). We also looked at the effect on endogenous SgII storage using immunofluorescence. Expression of the C-terminal domain of HID-1 tethered to the TGN (GFP-TGN38-C-term) in HID-1 KO PC12 cells rescued the levels of SgII to levels similar to WT PC12 cells, whereas expression of GFP didn’t rescue SgII levels (**Fig 4D**). Altogether these data suggest that the C-terminus of HID-1 (amino acids 687–788) contains the functional domain.

### Homozygous mutation within HID-1 C-terminus identified in human patients fail to rescue regulated secretion

**Fig 5:**
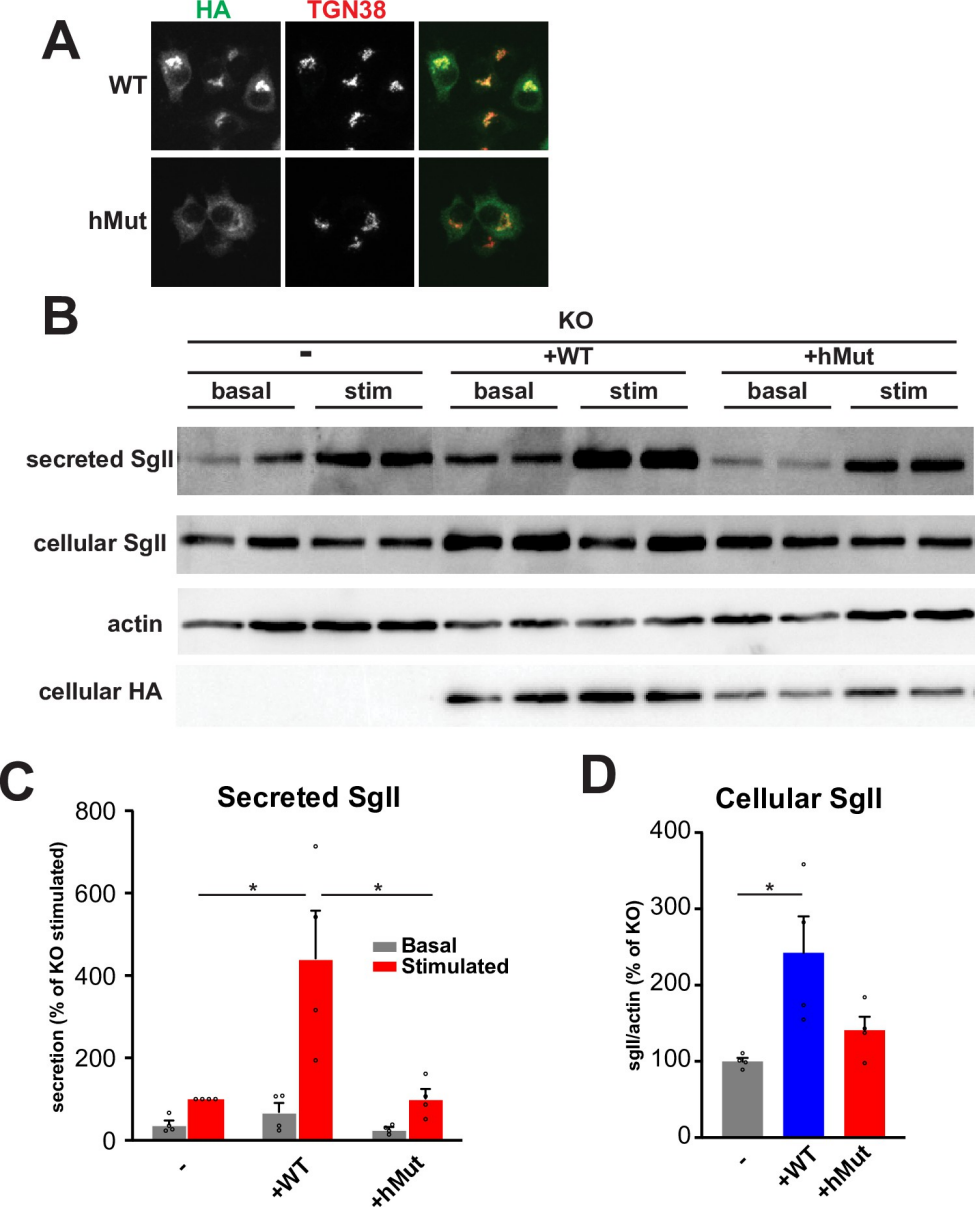
Homozygous mutation within HID-1 C-terminus identified in human patients fail to rescue regulated secretion. (**A**) HID-1 KO PC12 cells stably expressing WT HID-1 or the human mutant were immunostained for HA and TGN38. (**B-D**) Secretion assays were performed and quantified as described in **Fig 2** using HID-1 KO PC12 cells stably expressing WT or HID-1 human mutant. *, p < 0.05 relative to WT by one-way ANOVA (n = 4). The bar graphs indicate mean ± s.e. Scale bar indicates 10μm.

A homozygous mutation within HID-1 has been found in human patients with severe endocrine and neurological deficits [31,37]. This base-pair duplication leads to a frameshift at the end of HID-1 C-terminus (**S3 Fig**). The result of this mutation is predicted to substitute the last 15 amino acids (IWYDTDVKLFEIQRV) for 10 amino acids (CLVRHRREAV). Stable expression of this mutant in HID-1 KO PC12 cells led to proper TGN localization (**Fig 5A**). However, this mutant completely failed to rescue the secretion phenotype observed in HID-1 KO cells (**Fig 5B–5D**). These data suggest that a defect in LDCV formation is the underlying cause of the symptoms associated with this patient. Recent work also reported that mutations in HID-1 patients cause an early infantile encephalopathy with hypopituitarism [37] and further underscores that HID-1 plays a key role in the regulated secretory pathway. Unraveling its mechanisms of action might help us better understand diseases of the endocrine system and of the brain.

## Supporting information

S1 Fig (**A**) Protein sequence alignment of HID-1 N-terminus. (**B**) PC12 cells were transfected with the indicated GFP constructs. Cells were treated or not with saponin were fixed and immunostained for TGN38. Scale bars indicate 10μm.
(TIF)

S2 Fig (**A**) Genomic DNA sequence of rat HID-1. The predicted Cas9 cleavage site is indicated. (**B**) Indels (shown in red) detected in INS-1 HID-1 KO cells. (**C**) Indels (shown in red) detected in PC12 HID-1 KO cells. (**D-E**) HID-1 KO PC12 and INS-1 immunostained for endogenous HID-1. Scale bars indicate 10μm.
(TIF)

S3 Fig Protein sequence alignment of HID-1 C-terminus (103 amino acids) for the indicated species and for the human mutation. (TIF)

S4 Fig HA immunoblot showing expression level of indicated HID-1 constructs in stably transduced HID-1 KO PC12 cells. Tubulin is shown as a loading control.
(TIF)

S1 Table List of constructs and primers. (PDF)

S1 Raw images (PDF)
