# Supplementary figures and images for: Identification of the functional domain of the dense core vesicle biogenesis factor HID-1
Source: PLoS One. 2023 Sep 26;18(9):e0291977. doi: 10.1371/journal.pone.0291977 (PMC10522040; doi:10.1371/journal.pone.0291977)

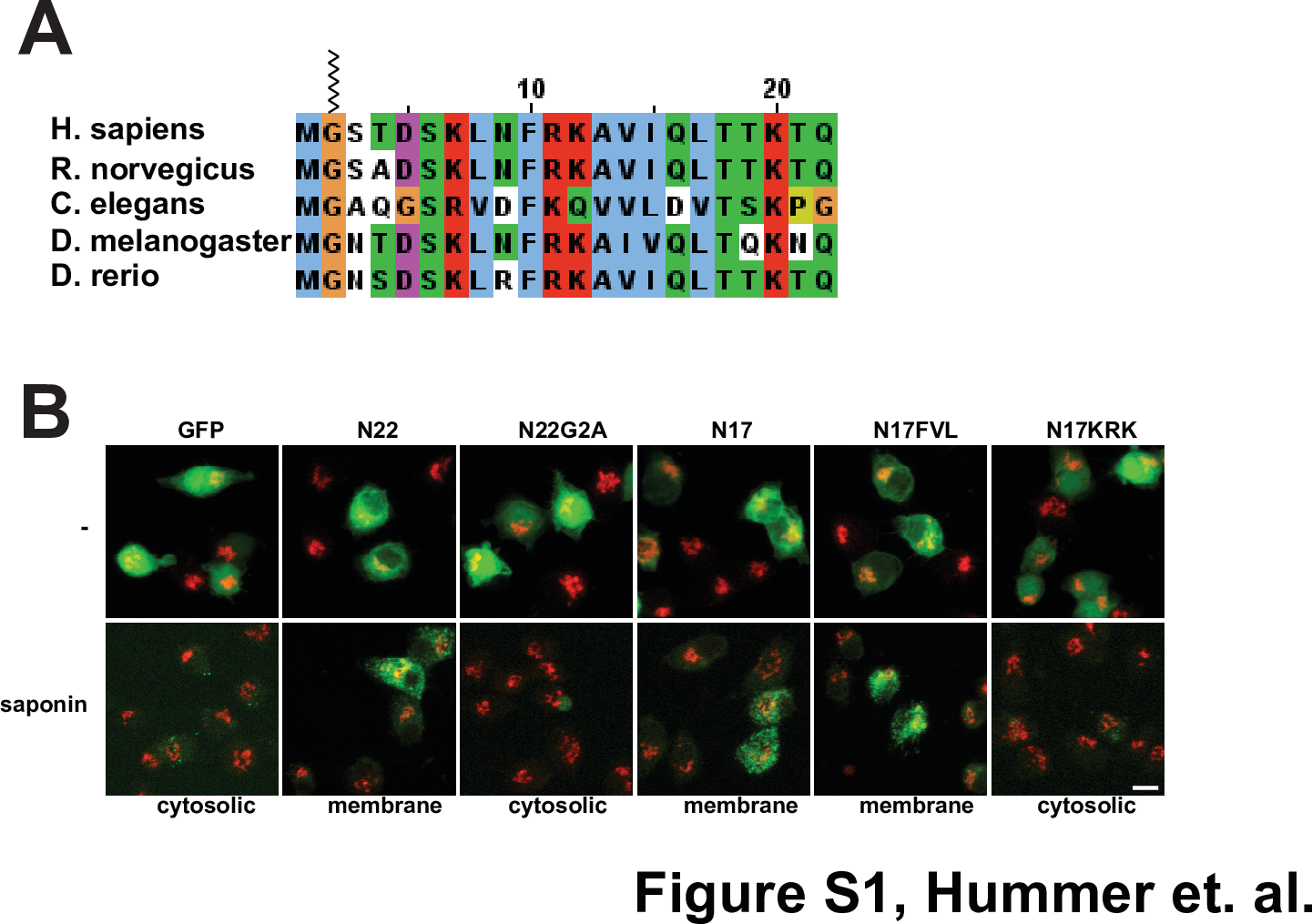

Supplement: S1 Fig — (A) Protein sequence alignment of HID-1 N-terminus. (B) PC12 cells were transfected with the indicated GFP constructs. Cells were treated or not with saponin were fixed and immunostained for TGN38. Scale bars indicate 10μm. (TIF) [file pone.0291977.s001.tif]

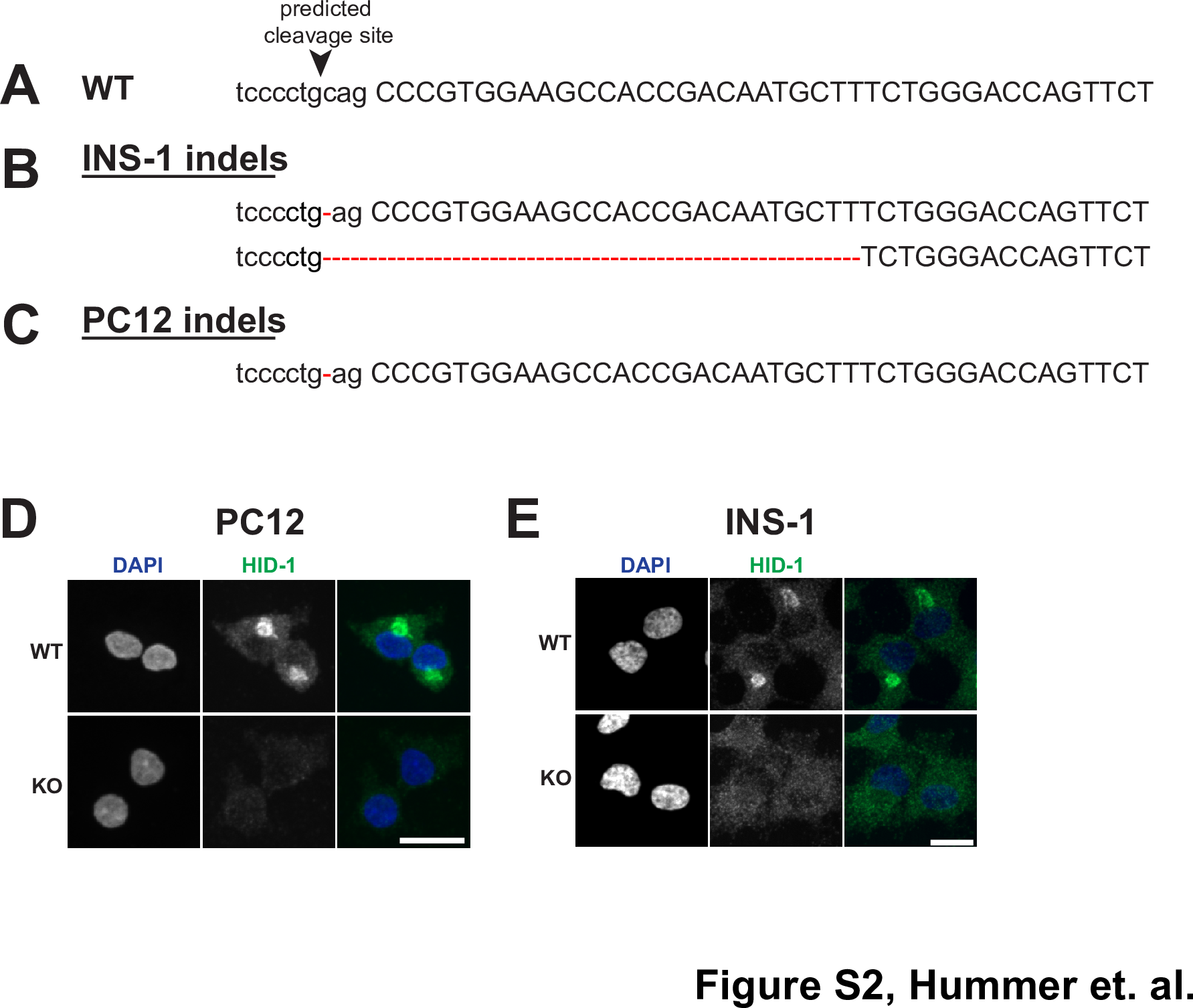

Supplement: S2 Fig — (A) Genomic DNA sequence of rat HID-1. The predicted Cas9 cleavage site is indicated. (B) Indels (shown in red) detected in INS-1 HID-1 KO cells. (C) Indels (shown in red) detected in PC12 HID-1 KO cells. (D-E) HID-1 KO PC12 and INS-1 immunostained for endogenous HID-1. Scale bars indicate 10μm. (TIF) [file pone.0291977.s002.tif]

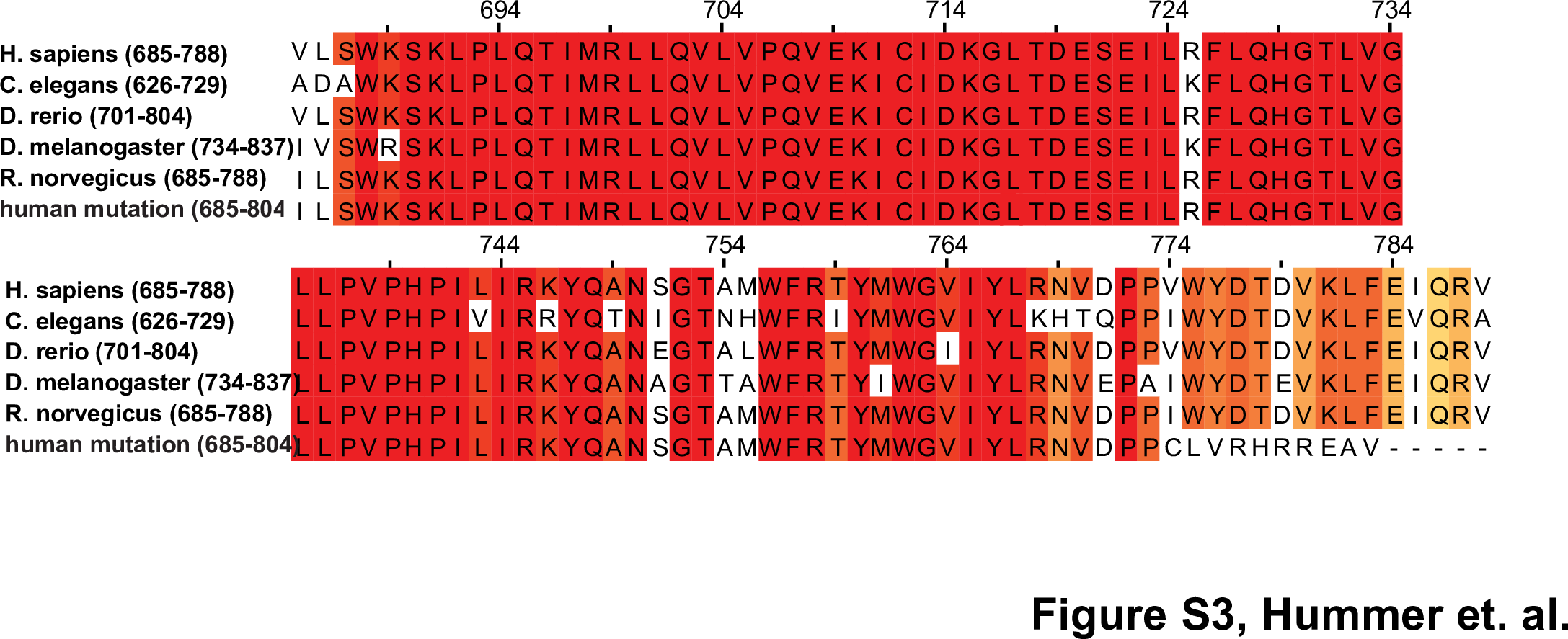

Supplement: S3 Fig — (TIF) [file pone.0291977.s003.tif]

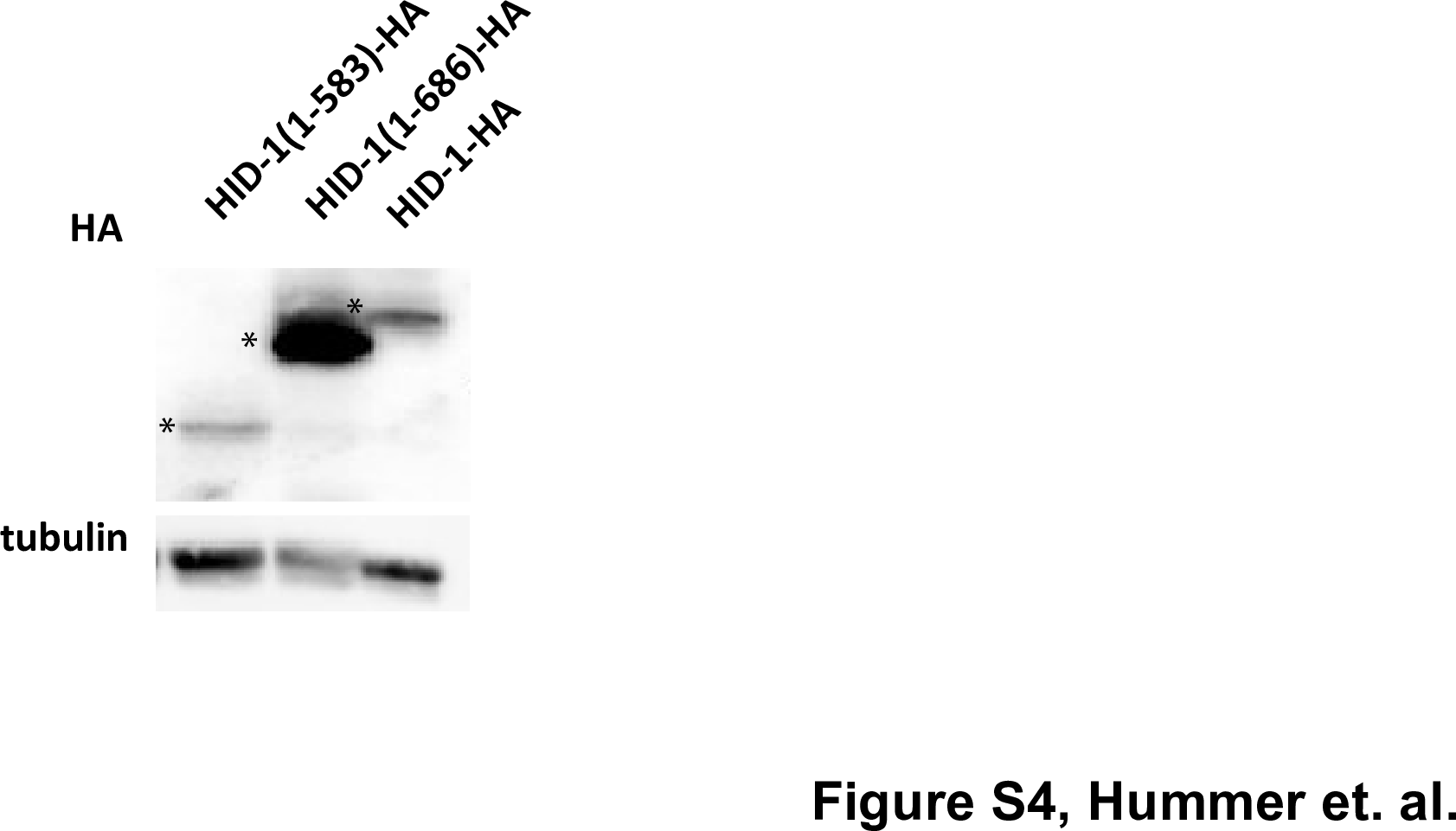

Supplement: S4 Fig — Tubulin is shown as a loading control. (TIF) [file pone.0291977.s004.tif]
