# Supplementary material for: Identification of the functional domain of the dense core vesicle biogenesis factor HID-1
Source: PLoS One. 2023 Sep 26;18(9):e0291977. doi: 10.1371/journal.pone.0291977 (PMC10522040; doi:10.1371/journal.pone.0291977)
Supplement: S1 Table — (PDF) [file pone.0291977.s005.pdf]

Table S1

Construct	Name	Forward sequence (5'-3')	Reverse sequence (5'-3')
pLenti-HID-1-HA	P1	ATGGGGTCTGCAGACTCC	CACCCGCTGGATCTCAAAGAG
pLenti-(G2A)HID-1-HA	P2	GTTACACCGGTGACCATGGCCTCTGCAGACTCC	AATCAGAATTCTTACACCCGCTGGATCTCAAAGAG
pLenti-(Δ22)HID-1-HA	P3	GTTACACCGGTGACCATGCCCGTGGAAGCCACCG	AATCAGAATTCTTACACCCGCTGGATCTCAAAGAG
pLenti-HID-1(hMutant)-HA	P4	CCTCCATAGAAGACACCGACTCTAGAGACCATGGGGTCTGCAGA	GAATTCAACAGCTTCACGTCGGTGTCGTACCAGACAGGGCGGGTCCACATTCCT
pCAGG-GFP-TGN38	P5	GTCTCATCATTTTGGCAAAGAATTCCACCATGAAGTGCCTTTTGTACT	CGGGACAGGGGCTCCGGTGTAAGCTTTAGGTTCAAACGTTGGTA
pCAGG-GFP-TGN38-HID-1	P6	AACGTTTGAACCTAAAGCTTTCCTGGAAGTCAAAACTGCC	TATGGGTAACCCCCAGATCCCTCGAGTCACACCCGCTGGATCTCAAAGA
pEGFP-N1-HID-1-N22	P7	GTTACGGATCCCACCATGGGGTCTGCAGACTCC	GTAACACCGGTCCCTGAGTCTTGGTAGTCAGCTGG
pEGFP-N1-HID-1-N22(G2A)	P8	GTTACGGATCCCACCATGGCCTCTGCAGACTCC	GTAACACCGGTCCCTGAGTCTTGGTAGTCAGCTGG
pEGFP-N1-HID-1-N17	P9	GTTACGGATCCCACCATGGGGTCTGCAGACTCCAAGCTGAACTTCCGG	GTAACACCGGTCCCTGCTGGATCTGCGCCTTCCGCTGGTTCAGCTTGG
pEGFP-N1-HID-1-N17(FVL)	P10	GTTACGGATCCCACCATGGGGTCTGCAGACTCCAAGCTGAACCAGCGG	GTAACACCGGTCCCTGCTGGATCTGCGCCTTCCGCTGGTTCAGCTTGG
pEGFP-N1-HID-1-N17(KRK)	P11	GTTACGGATCCCACCATGGGGTCTGCAGACTCCGCCCTGAACTTCGCC	GTAACACCGGTCCCAGCTGGATCACCGCGGCGGCGAAGTTCAGGGCGG
FUGW-HID-1(1-583)-HA	P14	GATCCCCGGGTACCGGTGACCATGGGGTCTGCAGACTCC	GTAACCCCCAGATCCGAATTCTGTCCTCCGCCGCC
FUGW-HID-1(1-686)-HA	P15	GATCCCCGGGTACCGGTGACCATGGGGTCTGCAGACTCC	GTAACCCCCAGATCCGAATTCGGACAAGATCCAATCTGATGTTGG
