# Supplementary material for: Identification of the functional domain of the dense core vesicle biogenesis factor HID-1
Source: PLoS One. 2023 Sep 26;18(9):e0291977. doi: 10.1371/journal.pone.0291977 (PMC10522040; doi:10.1371/journal.pone.0291977)
Supplement: S1 Raw images — (PDF) [file pone.0291977.s006.pdf]

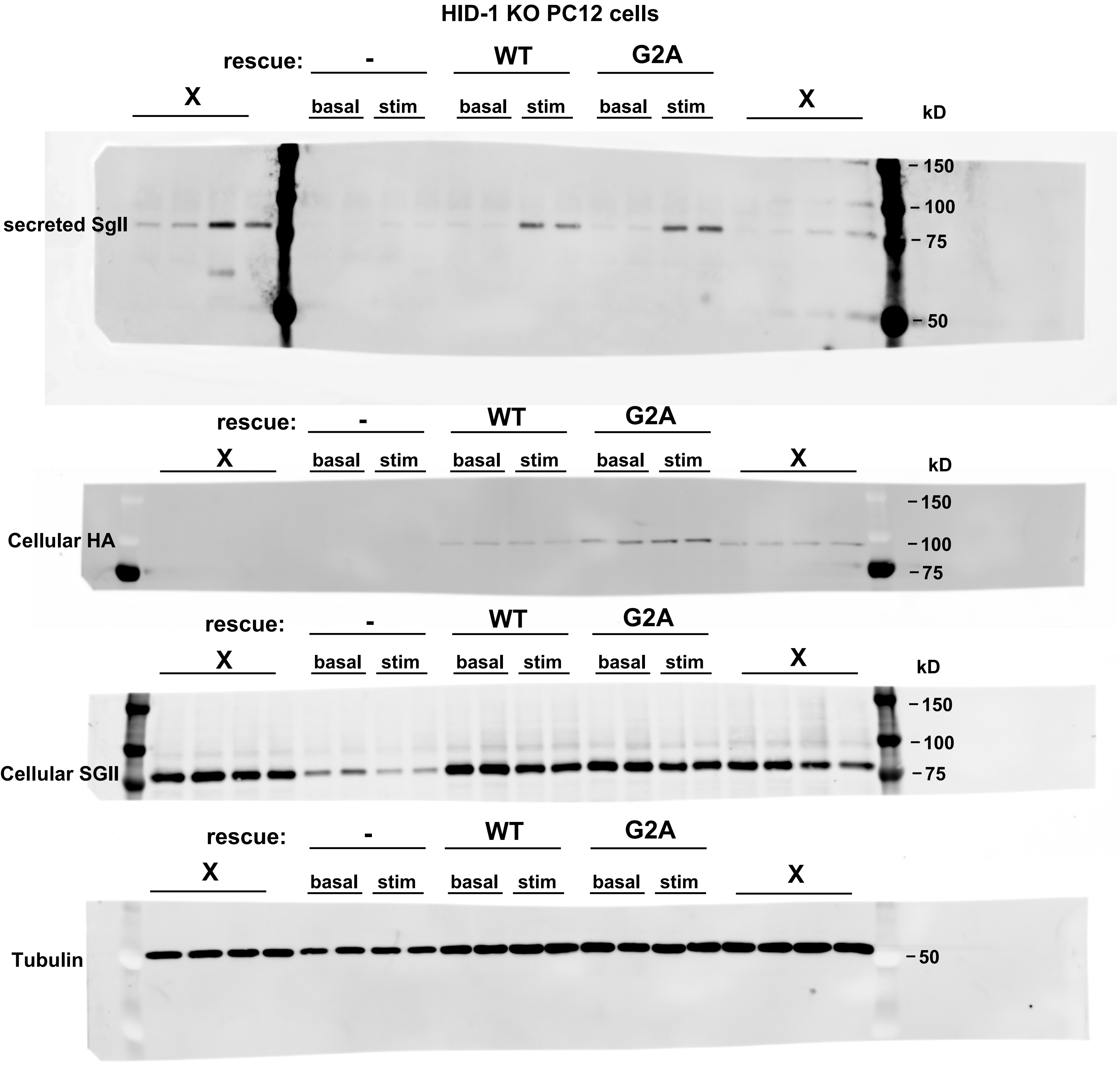

These images were taken on ProteinSimple FluorChem R

Figure 2a

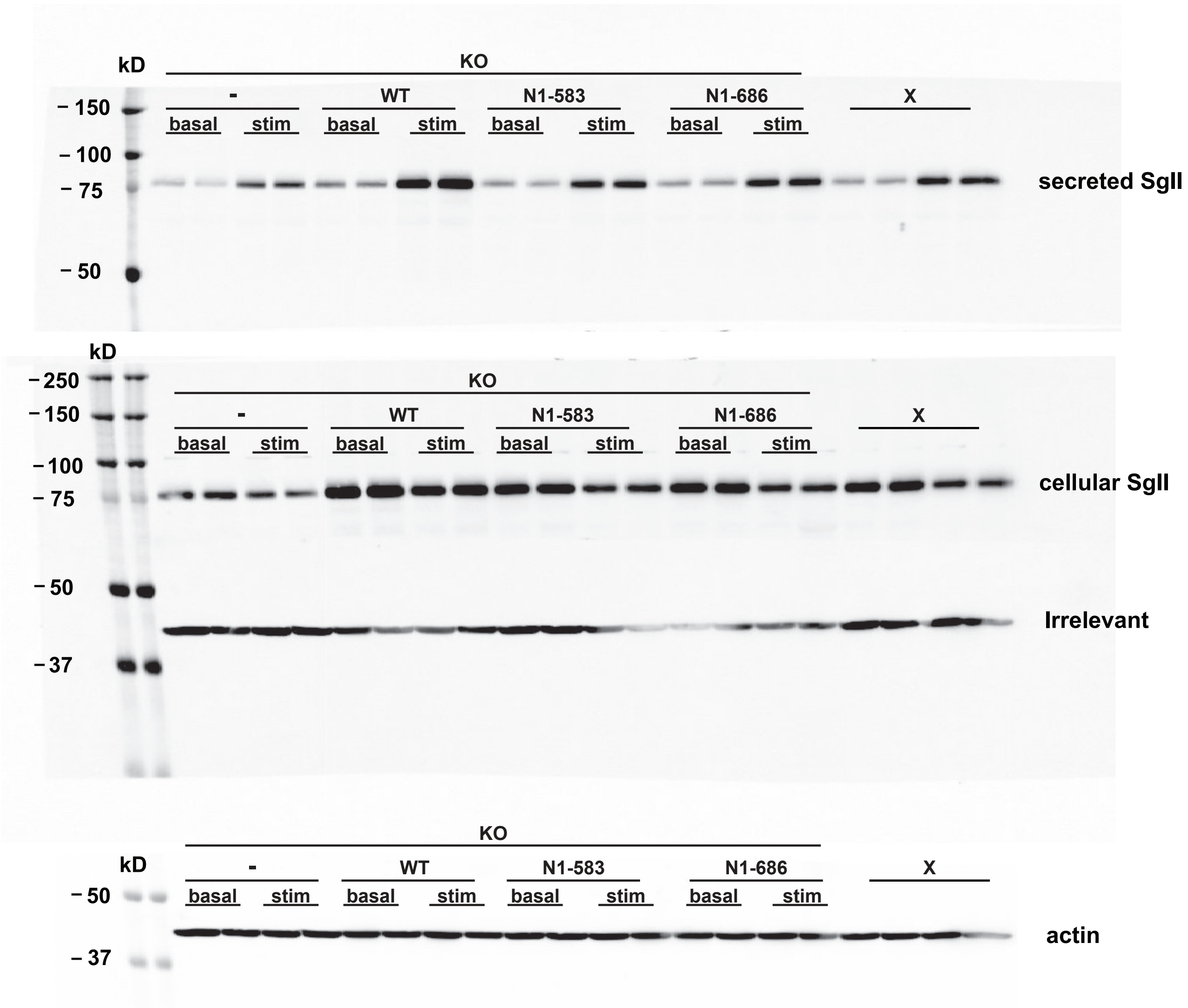

These images were taken on ProteinSimple FluorChem R

Figure 3a

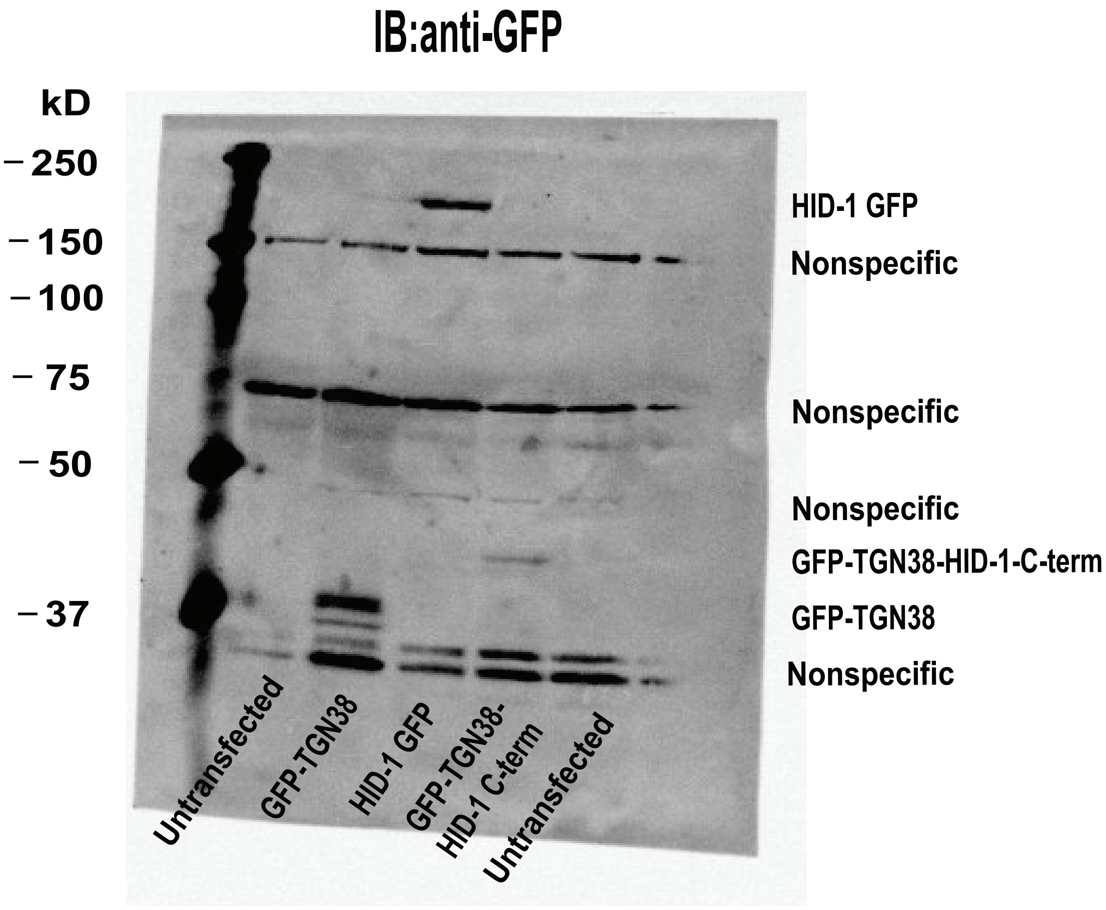

These images were taken on ProteinSimple FluorChem R

Figure 4A

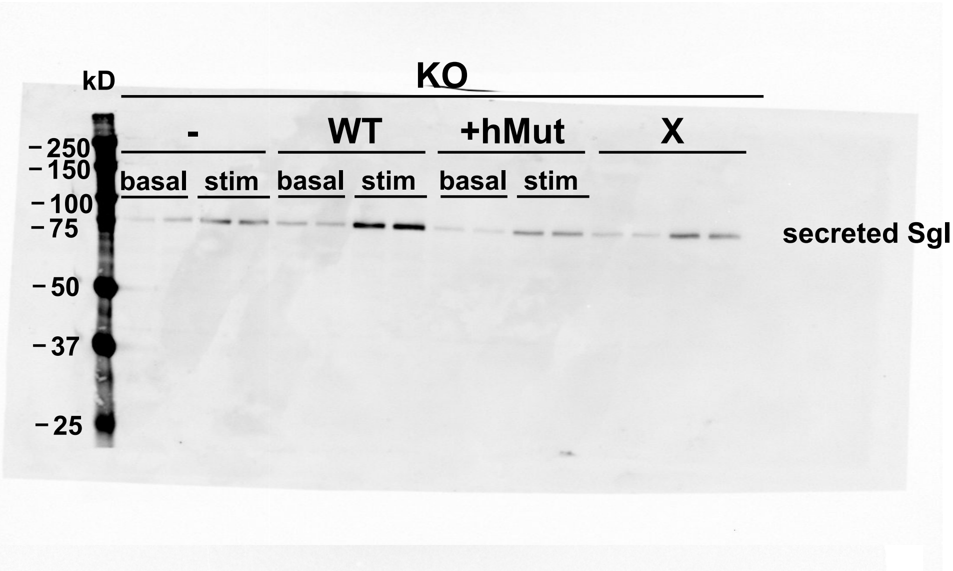

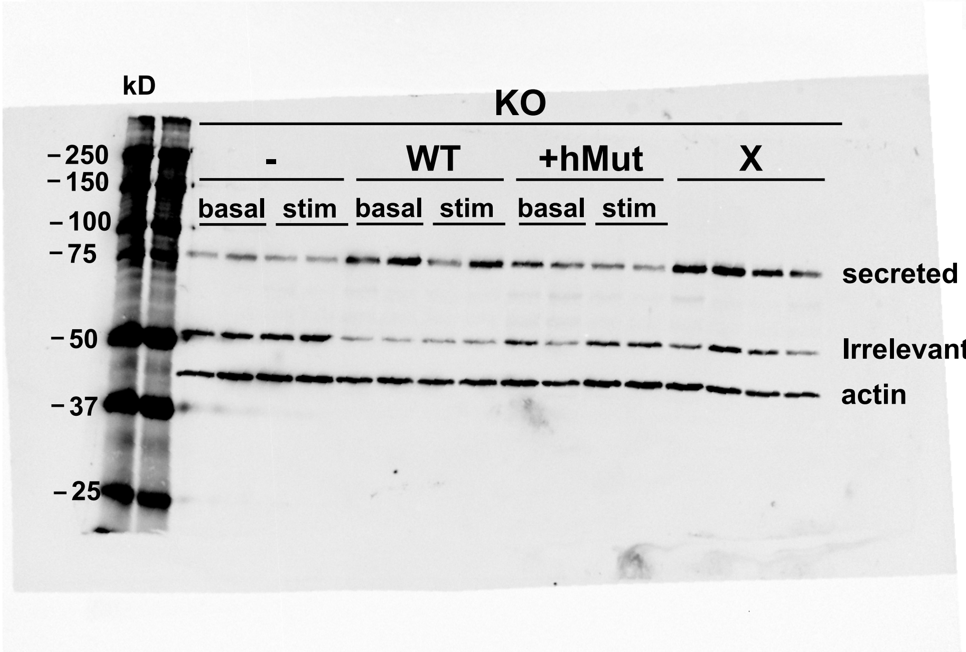

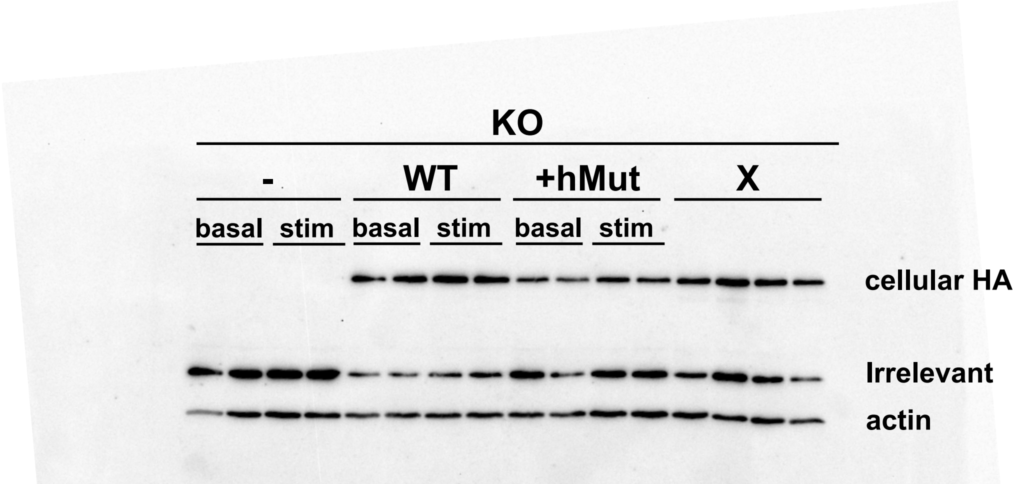

These images were taken on ProteinSimple FluorChem R

Figure 5b

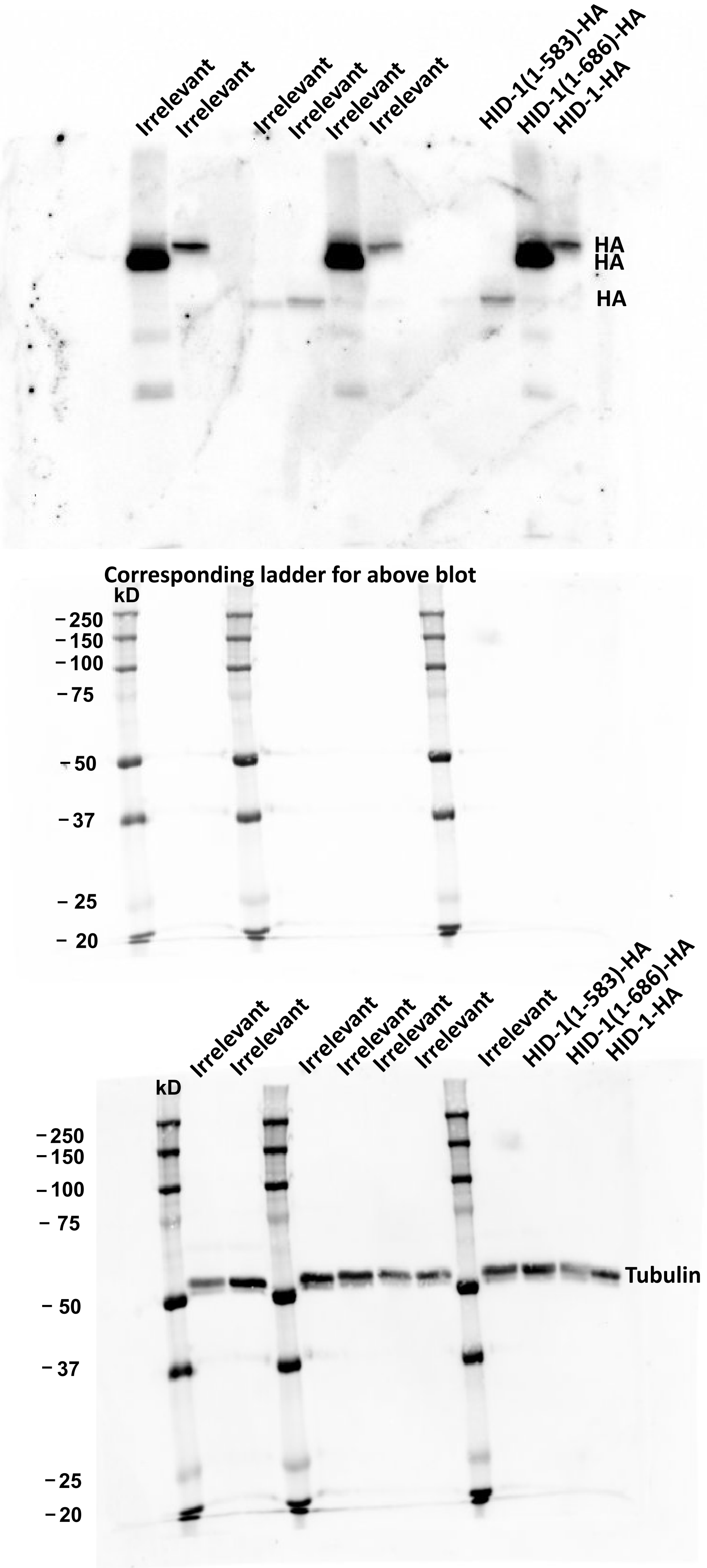

These images were taken on ProteinSimple FluorChem R

Figure S4
